# Molecular Analysis of the Genes Involved in Aroma Synthesis in the Species *S. cerevisiae*, *S. kudriavzevii* and *S. bayanus var. uvarum* in Winemaking Conditions

**DOI:** 10.1371/journal.pone.0097626

**Published:** 2014-05-22

**Authors:** Amparo Gamero, Carmela Belloch, Clara Ibáñez, Amparo Querol

**Affiliations:** Departamento de Biotecnología, Instituto de Agroquímica y Tecnología de Alimentos, CSIC, Valencia, Spain; University of Leicester, United Kingdom

## Abstract

The *Saccharomyces* genus is the main yeast involved in wine fermentations to play a crucial role in the production and release of aromatic compounds. Despite the several studies done into the genome-wide expression analysis using DNA microarray technology in wine *S. cerevisiae* strains, this is the first to investigate other species of the *Saccharomyces* genus. This research work investigates the expression of the genes involved in flavor compound production in three different *Saccharomyces* species (*S. cerevisiae*, *S. bayanus var. uvarum* and *S. kudriavzevii*) under low (12°C) and moderate fermentation temperatures (28°C). The global genes analysis showed that 30% of genes appeared to be differently expressed in the three cryophilic strains if compared to the reference strain (mesophilic *S. cerevisiae*), suggesting a very close cold adaptation response. Remarkable differences in the gene expression level were observed when comparing the three species, *S. cerevisiae*, *S. bayanus var. uvarum* and *S. kudriavzevii*, which will result in different aroma profiles. Knowledge of these differences in the transcriptome can be a tool to help modulate aroma to create wines with the desired aromatic traits.

## Introduction

Yeasts play a crucial role in the development of the so-called wine secondary aroma, with higher alcohols, acetate esters and ethyl esters being the main aromatic compounds that contribute to floral and fruity aroma [1]. Higher alcohols are synthesized from amino acids through transamination and decarboxylation reactions. These chemical reactions are carried out by amino acid permeases (codified by *GAP1*, *BAP2*, *MEP2*), transaminases (codified by *BAT1*, *BAT2*, *ARO8*, *ARO9*), decarboxylases (codified by *PDC1*, *PDC5*, *PDC6*, *THI3*, *ARO10*) and dehydrogenases (codified by *ADH1-7*, *SFA1*) [2]. Acetate esters are synthetised by a condensation between higher alcohols and acetyl-CoA.This reaction is mediated by acetyltransferases codified by genes *ATF1* and *ATF2* [3]. Ethyl esters are produced by condensation between ethanol and acyl-CoA, a reaction mediated by acyltransferases codified by genes *EHT1*, *EEB1* and *YMR210W* [3]. Besides, the effect of the esterases codified by *IAH1* and *TIP1* must be taken into account in the final concentration of both acetate and ethyl esters in wine [3]. *Saccharomyces* yeasts can also participate in primary aroma release through glycosidases [4]. Examples of the genes codifying glycosidases and glucanases are *BGL2, EXG1, SPR1* and the ORF *YIR007W* [5], [6].

During the winemaking process, ethanol, glycerol, acetic acid and acetaldehyde can be synthesised by yeasts. Ethanol decreases aroma perception by increasing the solubility of aromatic compounds in wine [7]. Acetic acid (volatile acidity) at a high concentration, as occurs in stuck and sluggish fermentations, confers wine an undesirable odor [8]. Acetaldehyde is obtained by pyruvate decarboxylation, and although it can be reduced to ethanol, a small quantity may remain and produce wine oxidation [8]. The genes codifying piruvate decarboxylases, aldehyde dehydrogenases and alcohol dehydrogenases are involved in the metabolism of acetaldehyde, acetic acid and ethanol.

The main yeasts responsible for wine production belong to *Saccharomyces* genus. *S. cerevisiae* is the most important species involved in winemaking, and closely related species *Saccharomyces bayanus var. uvarum* may also participate [9], [10]. From the oenological point of view, several properties of these *Saccharomyces* species differ. A comparison made between *S. bayanus var. uvarum* and *S. cerevisiae* revealed that the former is more cryotolerant and produces smaller acetic acid quantities [11]–[13]. Wines produced by *S. bayanus var. uvarum* strains have a higher aromatic intensity than those produced by *S. cerevisiae* [14], [15]. Specifically, *S. bayanus var. uvarum* generates larger amounts of 2-phenylethanol, 2-phenylethyl acetate and ethyl lactate [13], [16], [17]. In contrast, *S. bayanus var. uvarum* is less common and appears mainly in fermentations at low temperatures [18]. *S. kudriavzevii* has been isolated from decayed leaves in Japan [9], and recently from oak barks in Portugal [19] and Spain [20]. Although it is not involved in winemaking, *S. kudriavzevii* participates in the hybridization processes with other *Saccharomyces* species like *S. cerevisiae* or *Saccharomyces bayanus var. uvarum* [21]–[23].

Nowadays there is a winemaking trend which consists in lowering fermentation temperatures in order to improve the aromatic profile of wines. Previous studies have demonstrated that low-fermentation temperatures result in not only higher aroma retention, but also in reduced higher alcohols and volatile acidity, and in an increase of esters and fatty acids [1], [24], [25]. Other studies stress the importance of yeast species, or even strains, in aroma production [26]. However, low-fermentation temperatures have their disadvantages, including prolonged process duration and a higher risk of halted or sluggish fermentations [27]. As mentioned before, both *S. kudriavzevii* and *S. bayanus var. uvarum* are characterized as cryotolerant and they constitute a potential tool to carry out low-temperature fermentations efficiently [26]. After the genome sequence of *S. cerevisiae* was reported [28], many studies have been done on the genome-wide expression analysis using DNA microarrays to better understand winemaking processes [29], [30], temperature influence on growth or aroma production [31], [32], the genes involved in aroma production [33], a general or sugar stress response [34], [35], or the response to nitrogen depletion [36]. Despite several genome-wide expression studies in *S. cerevisiae* using DNA microarray technology, there is no equivalent information available on other species of the genus.

This research work focuses on the expression of the genes involved in the production of flavor compounds during winemaking in three different cryotolerant *Saccharomyces* strains of the species *S. cerevisiae*, *S. bayanus var. uvarum* and *S. kudriavzevii* at low and moderate fermentation temperatures.

## Materials and Methods

### Yeast strains

The yeast strains used in this study belong to different species from the genus *Saccharomyces*, and the commercial wine strains Lalvin T 73 and Fermol cryophile (*S. cerevisiae*), IFO 1802 (*S. kudriavzevii*) and CECT 12600 (*S. bayanus var. uvarum* var. *bayanus*).

### Fermentation and aroma analysis

Fermentative compounds and aroma data can be found in Gamero et al., 2013 [26].

### Comparative genomic hybridization (CGH)

*S. cerevisiae* strain S288C was used as a control for microarray hybridizations. Yeast strains were cultivated in 5 ml YPD (1% yeast extract, 2% peptone, 2% glucose), at 28°C for 24 h and DNA was isolated according to standard procedures [37].

The karyotyping experiments were carried out following the methodology proposed by [38]. All the experiments were performed using duplicate arrays, and Cy5-dCTP and Cy3-dCTP dye-swap assays were performed to reduce the dye-specific bias.

Array slides were scanned in an Axon GenePix 4100A scanner (Axon Instruments), and the images were analyzed using the GenePix Pro 6.0 software (Molecular Devices Corp., Union City, CA, USA). With the Acuity 4.0 software (Molecular Devices Corp., Union City, CA, USA), manually flagged bad spots were eliminated and the local background was subtracted before averaging the replicate features in the array. Log_2_ intensity ratios (M values) were then Median-normalized to correct for differences in the genomic DNA labeling efficiency between samples. The relative hybridization signal of each ORF was derived from the average of the two dye-swap hybridizations performed per strain. The normalized log_2_ ratio (M value) was considered a measure of the relative abundance of each ORF in relation to that of reference strain S288C. Deviations from the 1∶1 hybridization ratio were taken as being indicative of changes in the DNA copy number. Given that the variability usually observed between *Saccharomyces* genomes (either within laboratory strains or natural isolates) is much lower than this estimate, we interpreted the statistically significant depletions in the hybridization signal as ORF deletions.

The data discussed in this publication have been deposited in NCBI's Gene Expression Omnibus and are accessible through GEO Series accession number GSE52446 (http://www.ncbi.nlm.nih.gov/geo/query/acc.cgi?acc=GSE52446).

### Total RNA extraction and cDNA labeling with Cy3 and Cy5

Cells were collected by centrifugation (4000 rpm/min, 5 min) from two independent fermentations done at 12°C and 28°C at the beginning of stationary phase, and determined when 50% of the reducing sugars were consumed. The RNA extraction method was based on consecutive treatments with phenol-tris, phenol-chloroform (5∶1) and chloroform-isoamyl alcohol (24∶1), and a final precipitation with ethanol and sodium acetate [39]. RNA concentrations and purity were determined using a Nanodrop spectrophotometer ND-1000 (Nanodrop Technologies, Wilmington, DE, USA). RNA integrity was determined by electrophoresis in 1% agarose gel. 2–4 µg of total RNA from each sample was linearly amplified using the Low RNA Input Fluorescent Linear Amplification kit (Agilent Technologies, CA, USA). Then 2–3 ug of amplified cRNA were used as a template for cDNA synthesis. cDNA was marked indirectly with the “SuperScript Indirect cDNA Labeling System” (Invitrogen, San Diego, CA, USA). The fluorophores used were Cy3 and Cy5 mono-reactive Dye (Amersham GE Healthcare, Amersham, UK) and dye incorporation was monitored by a Nanodrop spectrophotometer.

### Microarrays hybridization, washing and scanning

A mixture of 200 to 300 pmol of the two labeled samples was concentrated in a Concentrator Plus (Eppendorf, Hamburg, Germany). Competitive hybridization was performed on a Yeast 6.4K Array with PCR-amplified ORFs of yeast S288c strain (Microarray Centre, UHN, Toronto, Ontario, Canada) in AHC hybridization chambers (ArrayIt Corporation, CA, USA) at 42°C overnight. The prehybridization solution contained 3X SSC, 0.1% SDS and 0.1 mg/ml BSA. The hybridization solution contained 5X SSC, 0.1% SDS and 0.1 mg/ml of salmon DNA. Microarrays were washed manually with different solutions containing distinct SSC 20X and SDS 10% concentrations (Sol.1: 2X SSC-0.1% SDS; Sol.2: 0.1X SSC-0.1% SDS; Sol.3: 0.1 SSC; Sol4: 0.01X SSC). The signal intensities of Cy3 and Cy5 were acquired with an Axon GenePix 4100A scanner (Molecular Devices, CA, USA) using the GenePix Pro v.6.1 software at a resolution of 10 µm.

### Microarray data analysis

Microarray data were derived from three independent experiments for cDNA hybridization. Raw data with global background subtraction were generated from GenePix pro 6.0. Analyses were done using the Acuity 4.0 software (Molecular Devices, CA, USA).Individual data sets were normalized to a log_2_ ratio value of 1. After normalization, data were filtered to remove the spots flagged as not found and were manually processed for print tip effect corrections. Only the spots with at least two replicates were considered. Finally, replicates were combined and their medians were calculated. The first cut-off was the selection of the genes presenting at least 2-fold log_2_ ratio values, according to the literature [31]–[36]. For these genes, a “GO terms” enrichment analysis was done using the GO Term Finder tool in the *Saccharomyces* Genome Database (http://www.yeastgenome.org/cgi-bin/GO/goTermFinder.pl). Regarding the statistics, a False Discovery Rate (FDR) analysis and a significance level of 99% (p value <0.01) were applied. Heat maps and hierarchical clustering were done using the Genesis software 1.7.6 (Graz University of Technology, Austria).

The data discussed in this publication have been deposited in NCBI's Gene Expression Omnibus and are accessible through GEO Series accession number GSE30778 (http://www.ncbi.nlm.nih.gov/geo/query/acc.cgi?acc=GSE30778).

## Results

A microarray analysis was carried out employing the RNA extracted from the cells harvested at the beginning of the stationary phase from the wine microfermentations done at 12°C and 28°C in *Tempranillo* must [26].

### Global analysis of genes presenting changes in expression

The two more divergent species used in this study were *S. cerevisiae* and *S. bayanus var. uvarum,* which display approximately 80% identity of coding and 74% identity of non coding sequences [40]. Hybridization of cDNA from the three *Saccharomyces* species was achieved under heterologous hybridization conditions in the *S. cerevisiae* microarrays. The hybridization conditions were previously tested by employing DNA-DNA microarrays, which showed that most genes of *S. bayanus var. uvarum* CECT 12600 (99.5%) and *S. kudriavzevii* IFO 1802 (98.7%) hybridize perfectly in the arrays and under the conditions employed in this study. Among the genes related to aroma synthesis, only *ILV5* and *PDA1* of *S. kudriavzevii* and *MUP3* of *S. bayanus var. uvarum* did not hybridize in the *S. cerevisiae* arrays employed in this study.

**Figure 1:**
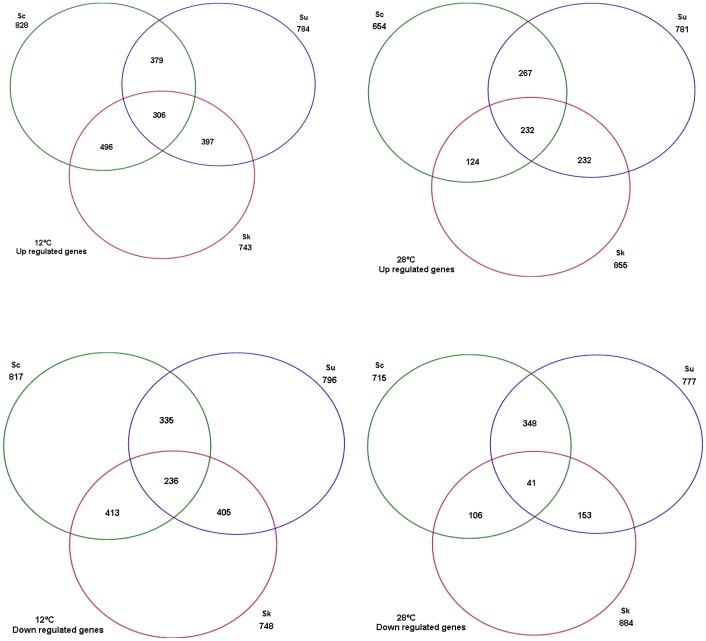
Global genetic expression analyses at 12°C and 28°C. Sc: commercial *Saccharomyces cerevisiae* Fermol Cryophile; Sb: *Saccharomyces bayanus var. uvarum* CECT 12600; Sk: *Saccharomyces kudriavzevii* IFO 1802.

Gene expression was determined at the beginning of the stationary phase in the fermentations carried out at 12°C and 28°C. The gene expression profiles of the three cryophilic strains used in this study (*S. cerevisiae* Fermol cryophile, *S. bayanus var. uvarum* CECT 12600 and *S. kudriavzevii* IFO 1802) were compared to the gene expression of reference mesophilic strain Lalvin T73. Only those genes with a fold change in expression of over 2 (positive or negative) in relation to *S. cerevisiae* Lalvin T73 were taken into account for further analyses. Figure 1 shows the amount of the up- and down-regulated genes found in each species in relation to Lalvin T73 at both fermentation temperatures. Aproximately 30% of the genes of the three cryophilic strains were differently expressed at 12°C or 28°C if compared to mesophilic *S. cerevisiae* Lalvin T73. The first point that stands out is the large number of up- and down-regulated genes shared by the three cryophilic species at 12°C, 306 and 236, respectively. However, a comparison made of the up- and down-regulated genes at 28°C revealed that only 77 up-regulated and 41 down-regulated genes were shared by the three cryophilic strains.

Go terms show the metabolic functions in which a significant number of up- or down-regulated genes are involved. Go terms were done with the up- and down-regulated genes for each species at both temperatures (Tables 1 and 2). No significant Go terms were found in any cryophilic strain among the up-regulated genes at 12°C. Conversely, the common down-regulated functions at 12°C among the cryophilic strains were observed. These include several basic metabolic pathways, such as catalytic activity and oxidoreductase activity. It is worth noting that *S. cerevisiae* Fermol Cryophile showed down-regulated metabolic functions in relation to transmembrane transport activity. Furthermore, *S. cerevisiae* Fermol Cryophile and *S. kudriavzevii* presented down-regulated genes in most of the genes involved in aryl-alcohol dehydrogenase activity (Table 1).

**Table 1:** Go terms for the down regulated genes at 12°C.

Fermol cryophile (Sc)	CECT 12600 (Su)	IFO 1802 (Sk)
3735. Structural constituent of ribosome	3824. Catalytic activity	3824. Catalytic activity
3824. Catalytic activity	16491. Oxidoreductase activity	4022. Alcohol dehydrogenase (NAD) activity
5353. Fructose transmembrane transporter activity	16614. Oxidoreductase activity, acting on CH-OH group of donors	16614. Oxidoreductase activity, acting on CH-OH group of donors
5355. Glucose transmembrane transporter activity	16616. Oxidoreductase activity, acting on the CH-OH group of donors, NAD or NADP as acceptor	16616. Oxidoreductase activity, acting on the CH-OH group of donors, NAD or NADP as acceptor
15144. Carbohydrate transmembrane transporter activity		16491. Oxidoreductase activity
15145. Monosaccharide transmembrane transporter activity		18456. Aryl-alcohol dehydrogenase activity
15149. Hexose transmembrane transporter activity		
15578. Mannose transmembrane transporter activity		
16491. Oxidoreductase activity		
16614. Oxidoreductase activity, acting on CH-OH group of donors		
16616. Oxidoreductase activity, acting on the CH-OH group of donors, NAD or NADP as acceptor		
18456. Aryl-alcohol dehydrogenase activity		
22857. Transmembrane transporter activity		
22891. Substrate-specific transmembrane transporter activity		
22892. Substrate-specific transporter activity		
51119. Sugar transmembrane transporter activity		
70011. Peptidase activity, acting on L-amino acid peptides		

GO terms obtained from *Saccharomyces* Genome Database http://www.yeastgenome.org/; Sc: *Saccharomyces cerevisiae*; Su: *Saccharomyces uvarum*; Sk: *Saccharomyces kudriavzevii*.

**Table 2:** Go terms for the up regulated genes at 28°C.

Fermol cryophile (Sc)	CECT 12600 (Su)	IFO 1802 (Sk)
3735. Structural constituent of ribosome	3735. Structural constituent of ribosome	No significant GO terms
3743.Translation initiation factor activity	3824. Catalytic activity	
5198. Structural molecule activity	5198. Structural molecule activity	
8135. Translation factor activity, nucleic acid binding	8135. Translation factor activity, nucleic acid binding	
15078. Hydrogen ion transmembrane transporter activity	16491. Oxidoreductase activity	
16491. Oxidoreductase activity		

GO terms obtained from *Saccharomyces* Genome Database http://www.yeastgenome.org/; Sc: *Saccharomyces cerevisiae*; Su: *Saccharomyces uvarum*; Sk: *Saccharomyces kudriavzevii*.

However, the common up-regulated functions at 28°C were observed between the *S. cerevisiae* Fermol Cryophile and *S. bayanus var. uvarum* strains, among which the structural functions of the ribosome, nucleic acid binding, translation factor activity and oxidoreductase activity were found. In addition, *S. cerevisiae* presented an up-regulation in transmembrane transport, and *S. bayanus var. uvarum* did so in catalytic activity (Table 2). No significant GO terms were found for *S. kudriavzevii.* Finally at 28°C, no significant Go terms were observed in any cryophilic strain among the down-regulated genes.

### Analysis of the expression of genes related to aroma production

**Figure 2:**
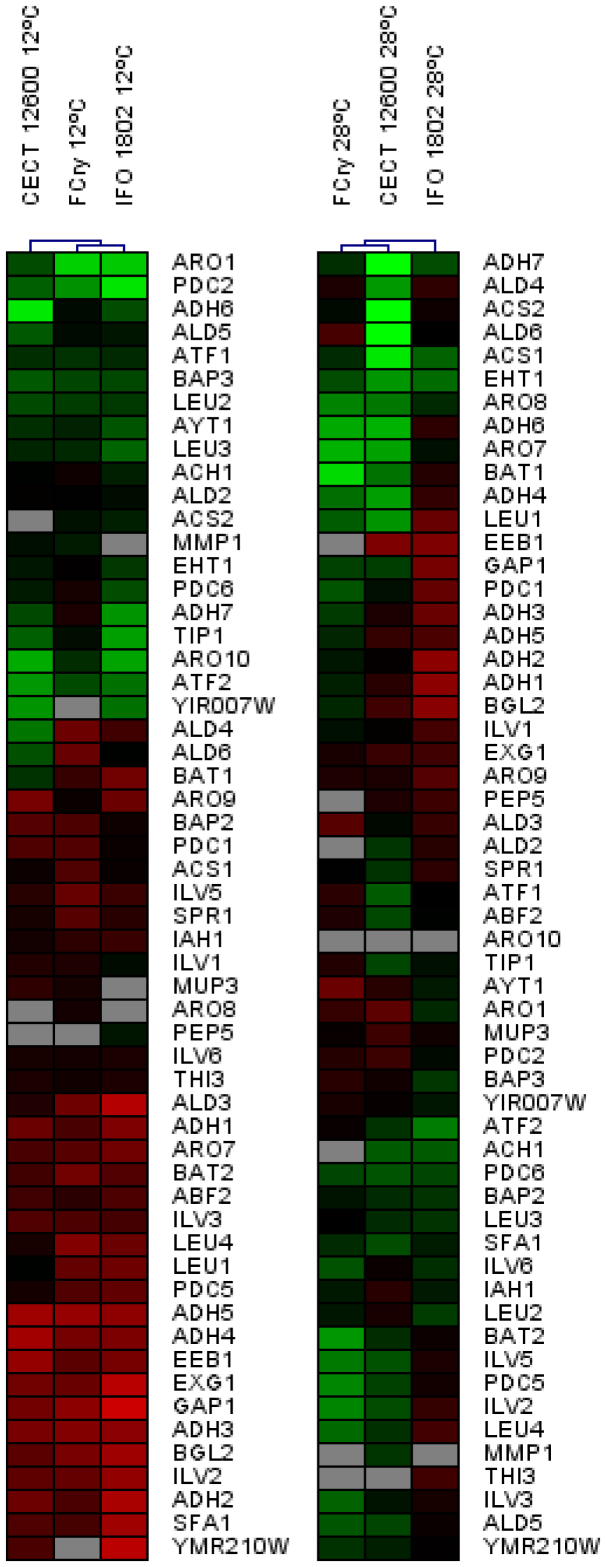
Heat maps depicting the level of expression of the genes related to flavour formation at 12°C and 28°C. Green: up-regulation; red: down-regulation; black: no changes in expression; gray: no hybridization.

The expression level of the genes involved in amino acids, higher alcohols, acetate esters, ethyl esters, ethanol, acetaldehyde and acetate metabolism, and the enzymes involved in wine primary aroma release, appear in Figure 2.

The hierarchical clustering of the gene expression at 12°C of the genes involved in aroma formation showed that the expression profiles of *S. cerevisiae* and *S. kudriavzevii* were closer than that of *S. bayanus var. uvarum*. Conversely, the expression profile of *S. cerevisiae* was similar to that of *S. bayanus var. uvarum* at 28°C, but differed from the gene expression profile of *S. kudriavzevii*.

According to gene expression, two groups of genes were clearly seen at 12°C (Figure 2): the genes up-regulated in the three species (green in the upper part of the heat map) and the genes down-regulated in the three species (red in the lower part of the heat map). By taking into account only the genes presenting an at least 2-fold change in expression in comparison with the reference strain, it is possible to divide the genes relating to aroma production into twelve different clusters.

In contrast, no clear groups were observed at 28°C. When bearing in mind only the genes presenting an at least 2-fold change in expression in comparison to the reference strain, it is possible to divide the genes relating to aroma production into eleven different clusters.

### Analysis of the genes presenting the same changes in expression in *S. cerevisiae*, *S. bayanus var. uvarum* and *S. kudriavzevii*

In this part of the work, the objective was to compare the differences in gene expression of the three cryotolerant *Saccharomyces* strains of the species *S. cerevisiae* (Fermol cryophile), *S. bayanus* and *S. kudriavzevii* employing mesophilic *S. cerevisiae* Lalvin T.73 as a reference.

**Figure 3:**
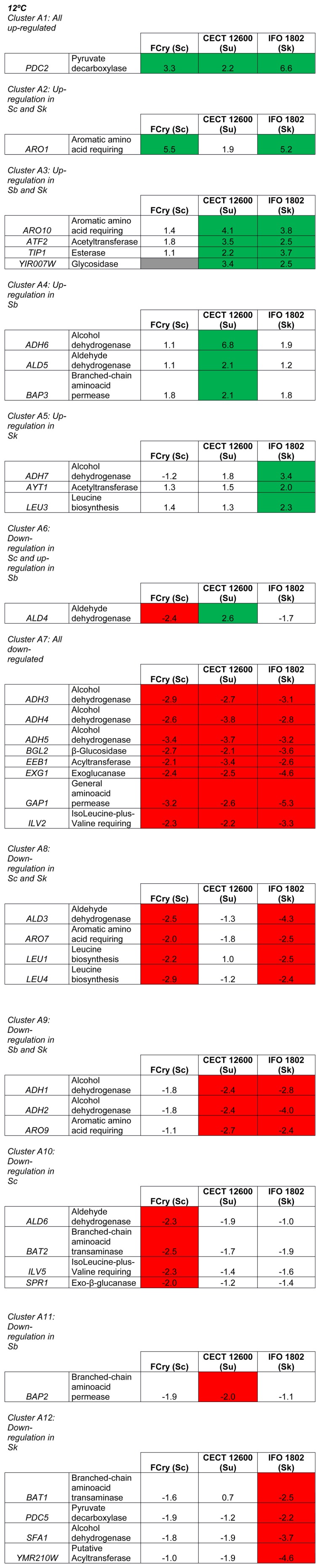
Clusters according to different changes in expression. *S. cerevisiae* (Sc), *S. bayanus var. uvarum* (Su) and *S. kudriavzevii* (Sk) at 12°C.

In the fermentations carried out at 12°C, only gene *PDC2* (pyruvate decarboxylase) appeared to be up-regulated in the three strains included in this study (Figure 3, Cluster A1). Conversely, larger numbers of genes were down-regulated in the three species (Figure 3, Cluster A7). These genes were some alcohol dehydrogenases (*ADH3-5*), genes codifiying enzymes related to wine primary aroma release (*BGL2* and *EXG1*), and some genes related to amino acids metabolism (the general amino acid permease codifyed by *GAP1* and *ILV2*, involved in branched-chain amino acids synthesis). The metabolisms of amino acids and ethanol/acetaldehyde were also affected.

**Figure 4:**
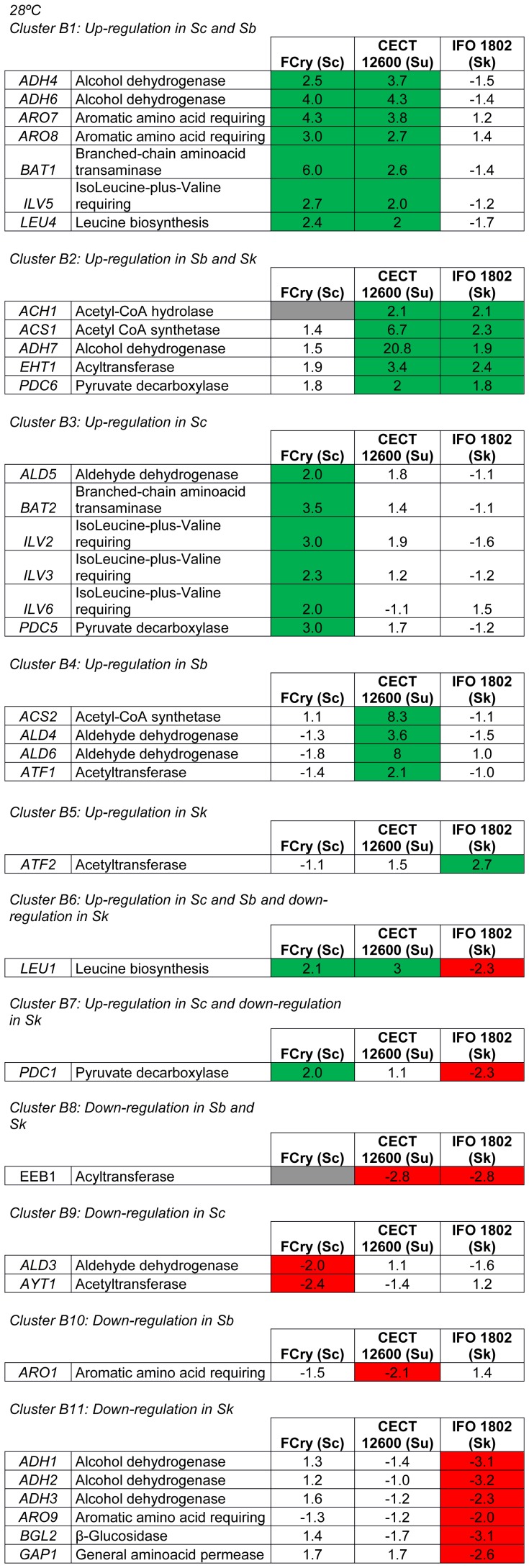
Clusters according to different changes in expression. *S. cerevisiae* (Sc), *S. bayanus var. uvarum* (Su) and *S. kudriavzevii* (Sk) at 28°C.

In the fermentations carried out at 28°C (Figure 4), the three criotolerant strains showed no gene presenting the same changes in expression comparing to the reference strain.

### Analysis of the genes presenting the same changes in expression in *S. cerevisiae* and *S. bayanus var. uvarum*

In the fermentations done at 12°C, *S. cerevisiae* and *S. bayanus var. uvarum* did had not have any gene that presenteding the same changes in expression if comparedwith respect to the reference strain. In the fermentations carried out at 28°C, several genes were up-regulated in *S. cerevisiae* and *S. bayanus var. uvarum* (Figure 4, Cluster B1). These genes codify alcohol dehydrogenases (*ADH4, ADH6*), a transaminase (*BAT1*), and also the enzymes involved in aromatic and branched-chain amino acids synthesis (*ARO7, ARO8, ILV5, LEU4*).

### Analysis of the genes presenting the same changes in expression in *S. cerevisiae* and *S. kudriavzevii*

In the fermentations at 12°C (Figure 3), *S. cerevisiae* and *S. kudriavzevii* presented an up-regulation in gene *ARO1*, which is involved in the synthesis of aromatic amino acids (Figure 3, Cluster A2). At this temperature, these two species shared several down-regulated genes (Figure 3, Cluster A8), and these genes were *ALD3*, codifying an aldehyde dehydrogenase, and some genes involved in branched-chain and aromatic amino acids synthesis (*ARO7, LEU1, LEU4*). In the fermentations performed at 28°C (Figure 4), *S. cerevisiae* and *S. kudriavzevii* had no gene which presented the same changes in expression if compared to the reference strain.

### Analysis of the genes presenting the same changes in expression in *S. bayanus var. uvarum* and *S. kudriavzevii*

In the fermentations at 12°C, *S. bayanus var. uvarum* and *S. kudriavzevii* (the two typical criotoleranst species), up-regulation was seen in some genes that presented different functions (Figure 3, Cluster A3), such as the synthesis of aromatic amino acids (*ARO10*), and acetyltransferase, esterase and glycosidase activities (*ATF2, TIP1* and *YIR007W*, respectively).

However at the same temperature, it is possible to see in Cluster A9 (Figure 3) that *S. bayanus var. uvarum* and *S. kudriavzevii* presented a down-regulation in two genes codifying alcohol dehydrogenases (*ADH1-2*) and in one gene involved in the synthesis of aromatic amino acids (*ARO9*). In the fermentations done at 28°C, Cluster B2 (Figure 4) shows several up-regulated genes in both *S. bayanus var. uvarum* and *S. kudriavzevii*, such as *ACH1* and *ACS1* involved in the acetate synthesis and degradation, respectively, and the degradation of acetate in *S. bayanus var. uvarum* was more highly expressed than in *S. kudriavzevii*. In addition, the up-regulation of alcohol dehydrogenase gene *ADH7*, acyltransferase gene *EHT1* and pyruvate decarboxylase gene *PDC6* also occurred. The acyltransferase codified by *EEB1* appeared to be down-regulated in both *S. bayanus var. uvarum* and *S. kudriavzevii* (Figure 4, Cluster B8). Finally, it is worth mentioning the high expression of *PDC6*.

### Analysis of the genes presenting changes in expression exclusively in the cryotolerant *S. cerevisiae strain.*

Interestingly, in the fermentation at 12°C, no genes were exclusively up-regulated in *S. cerevisiae* (Fermol cryophile). Nevertheless, several genes showed a down-regulation of about 2–2.5 fold in this species (Figure 3 Cluster A10), such as aldehyde dehydrogenase gene *ALD6* and some genes involved in the branched-chain amino acids metabolism (*BAT2, ILV5*). Besides, *S. cerevisiae* showed a down-regulation in exo-β-glucanase gene *SPR1*.

In the fermentation at 28°C, several genes appeared to be exclusively up-regulated in *S. cerevisiae* (Figure 4 Cluster B3), such as some of the genes involved in branched-chain amino acids metabolism (*BAT2, ILV2-3, ILV6*), and some genes related to acetaldehyde metabolism (*ALD5* and *PDC5*). At 28°C, *S. cerevisiae* also presented two genes that were exclusively down-regulated (Figure 4, Cluster B9), aldehyde dehydrogenase gene *ALD3* and acetyltransferase *AYT1*.

### Analysis of the genes presenting changes in expression exclusively in *S. bayanus var. uvarum*

In the fermentation at 12°C, some genes appeared to be exclusively up-regulated in *S. bayanus var. uvarum* (Figure 3, Cluster A4), such as alcohol dehydrogenase gene *ADH6* and branched-chain amino acid permease gene *BAP3*. In addition, the down-regulation of branched-chain amino acid permease gene *BAP2* appeared (Figure 3, Cluster A11).

In the fermentation performed at 28°C, some genes were exclusively up-regulated in *S. bayanus var. uvarum* (Figure 4, Cluster B4), such as *ACS2* and aldehyde dehydrogenase genes *ALD4* and *ALD6*. Finally, *S. bayanus var. uvarum* only presented one gene that was exclusively down-regulated at 28°C (Figure 4, Cluster B10). This gene is involved in the synthesis of aromatic amino acids (*ARO1*).

### Analysis of the genes presenting changes in expression exclusively in *S. kudriavzevii*

In the fermentation at 12°C, some genes were exclusively up-regulated in *S. kudriavzevii* (Figure 3, Cluster A5), such as alcohol dehydrogenase gene *ADH7,* gene *LEU3* involved in leucine biosynthesis, and acetyltransferase gene *AYT1*. Several genes were exclusively down-regulated in *S. kudriavzevii* (Figure 3, Cluster A12), branched-chain amino acid transaminase gene *BAT1* and alcohol dehydrogenase *SFA1*. The down-regulation of pyruvate decarboxylase gene *PDC5,* and in the putative acyltransferase codified by *YMR210W,* was also observed. In the fermentation at 28°C, only acetyltransferase gene *ATF2* appeared to be exclusively up-regulated in *S. kudriavzevii* (Figure 4, Cluster B5). The down-regulation in some alcohol dehydrogenases (*ADH1-3*), *ARO9*, involved in aromatic amino acids metabolism, and general aminoacid permease gene *GAP1* (Figure 4 cluster B11), were also observed. Finally, this species showed down-regulation in *BGL2*, which codified a β-glucosidase and was involved in primary aroma release (Figure 4, cluster B11).

### Other genes presenting changes in expression

Other genes were found to be up- or down-regulated depending on the species. In the fermentations done at 12°C, aldehyde dehydrogenase gene *ALD4* was down-regulated in *S. cerevisiae* (Fermol cryophile), but was up-regulated in *S. bayanus var. uvarum*, and no change in expression was observed for *S. kudriavzevii* (Figure 3, Cluster A6).

At 28°C, one gene involved in the biosynthesis of leucine (*LEU1*) was up-regulated in *S. cerevisiae* and *S. bayanus var. uvarum*, whereas this gene was down-regulated in *S. kudriavzevii* (Figure 4, Cluster B6). Finally, pyruvate decarboxylase gene *PDC1* was up-regulated in *S. cerevisiae* and was down-regulated in *S. kudriavzevii* (Figure 4, Cluster B7).

## Discussion

Functional genomic approaches, such as microarray technology, are powerful tools to analyze gene expression at the whole genome level, and provide a comprehensive view of yeast physiology [41]–[43]. However, yeast secondary metabolism is a complex network of biochemical pathways which, although well mapped from a biochemical viewpoint, is not well understood in terms of its physiological roles and genetic and biochemical regulation [34].

The genetic profile of the yeast used when carrying out the alcoholic fermentation, mainly of the *Saccharomyces* genus, is obviously important in the formation of the metabolites conferring specific flavors to wine [44]. Besides, other factors like temperature can influence the aromatic quality of wine. Several authors have observed that low-temperature fermentations lead to greater aroma retention, a drop in higher alcohols and volatile acidity, and to an increase in volatile esters [1], [24], [25], [27], [45]. However, other studies suggest that the way in which fermentation temperature affects the wine aroma profile is highly dependent on the strain that carries out the process [26].

The expression of the genes related to aroma production was determined at the beginning of the stationary phase since the most active period of aroma compound accumulation appears to occur in earlier fermentation stages [34], [44]. The species studied in the present research were selected for their remarkable aroma production during wine microfermentations in *Tempranillo* must at 12°C and 28°C, and also for their adaptation to ferment well at low temperature [26]. The use of *S. cerevisiae* microarrays to hybridize different *Saccharomyces* species did not pose a problem since the *Saccharomyces* species evaluated in this study presented high percentages of genetic homology, and heterologous hybridization conditions were employed to increase hybridization efficiency. Only the genes that presented changes in expression of at least 2-fold have been mentioned in this research work given their potential impact on aroma production during fermentation. The selection of this cut-off is common practice and has been used in several global analysis studies in the past to investigate gene expression [31], [33]–[36]. The global analysis of the genes showed that 30% of the genes appeared to be differently expressed in the cryophilic strains if compared to the mesophilic reference strain, and that the three cryophilic strains shared many similarities in gene expression at 12°C, suggesting a very close cold adaptation response. Conversely, the list of the shared genes presenting changes in expression at 28°C was very limited.

A previous study, which consisted in microfermentations in *Tempranillo* must with the same strains employed in this research work, showed that at 12°C, *S. bayanus var. uvarum* excelled in higher alcohol and acetate ester production, whereas *S. cerevisiae* did so in ethyl esters synthesis. In addition, both strains yielded a large amount of acetaldehyde. Yet at 28°C, the production of higher alcohols and acetate esters by *S. cerevisiae* was remarkable, as was the acetate ester and acetic acid synthesis carried out by *S. kudriavzevii* [26]. Finally, *S. bayanus var. uvarum* yielded a large amount of acetaldehyde at 28°C. A comparison made between the transcriptomic data obtained in this research work and the aforementioned chemical data indicates certain correlations. Higher alcohol levels produced by different species can be explained by gene expression at both temperatures, whereas it is not possible to correlate ester amounts with gene expression data in all cases. In these cases, differences might be due to differences in the enzyme activities involved in the corresponding pathway or other explanations can be hypothesized. For instance, alcohol dehydrogenases *ALD4* and *ALD5* (involved in acetaldehyde conversion into acetate) were up-regulated in the *S. bayanus var. uvarum* strain at 12°C. One possible explanation for the low acetate levels detected in the wines produced by this strain is that part of this compound is used for ethyl acetate production since this species produces the largest ethyl acetate amount at this temperature.

Some genes that perform the same enzymatic function might be more important in aroma formation than others, and must also be taken into account to analyze the correlation between chemical data and the transcriptome. For example, the *S. bayanus var. uvarum* and *S. kudriavzevii* strains at 28°C presented an up-regulation in acyltranferase *EHT1* and a down-regulation in acyltranferase *EEB1* (the genes involved in ethyl esters formation). The low ethyl ester production in the *S. bayanus var. uvarum* and *S. kudriavzevii* strains as compared to the reference strain suggests that acyltransferase *EEB1* is more important in the production of these aromatic compounds than *EHT1*, which has been previously described [34]. Besides, both acyltransferases have been related to esterase activity [46]. Likewise, our data confirms that *ADH4* has not a major role in the interconversion of ethanol and acetaldehyde as *ADH1* is the main gene responsible for this transformation [47]. In higher alcohol synthesis, different families of amino acids are involved; branched-chain amino acids valine and leucine are necessary for isobutanol and isoamyl alcohol production, respectively, whereas aromatic amino acid phenylalanine is required for 2-phenylethanol synthesis. The up-regulation of the genes codifying the permeases, transaminases and other enzymatic activities involved in branched-chain amino acids metabolism was observed in the *S. bayanus var. uvarum* and *S. cerevisiae* strains at 28°C. Furthermore according to the chemical data, higher levels of isobutanol and/or isoamyl alcohol were found in *S. cerevisiae* strain (Fermol cryophile), at 28°C than in the reference strain [26]. However, no increase in any of these compounds was observed in the *S. bayanus var. uvarum* strain at this temperature [26], although this strain presented up-regulated alcohol dehydrogenases. The discrepancy found between the chemical data and the transcriptome in *S. bayanus var. uvarum* at 28°C may be due to the utilization of 2-phenylethanol to produce the corresponding acetate. The larger amount of this acetate detected in *S. bayanus var. uvarum* at 28°C if compared to the reference strain supports this hypothesis [26]. The relevant production of 2-phenylethanol and the corresponding acetate, phenylethyl acetate, is a typical trait of the *S. bayanus var. uvarum* species [16]–[18], [48]. Conversely, the down-regulation of the gene codifying enzymes involved in branched-chain amino acids metabolism in the *S. cerevisiae* strain at 12°C and the *S. kudriavzevii* strain at 28°C coincided with isobutanol and/or isoamyl alcohol production [26]. Furthermore, the up-regulation of the genes codifying transaminases and other enzymes relating to aromatic amino acids metabolism were found in *S. cerevisiae* and *S. bayanus var. uvarum* at 28°C, which correlated with high 2-phenylethanol production.

In our study, the best acetate ester producers at 12°C, *S. bayanus var. uvarum* and *S. kudriavzevii* [26], presented an up-regulation in acetyltransferase gene *ATF2*, whereas at 28°C, only in the case of *S. kudriavzevii* it was possible to find a correlation between genetic and phenotypic data. Previous studies have also struggled to find correlations when analyzing the correspondence between *ATF1* and *ATF2* and the acetate ester levels in fermentations conducted by *S. cerevisiae* at 13°C and 25°C [32]. Oher authors have observed that mutants and transformants, which overproduce certain higher alcohols, showed a clearly increased synthesis of the respective acetate esters [49], [50], whereas other works have affirmed that ester synthesis cannot be explained solely by higher alcohol availability since high oxygen and unsaturated fatty acid levels are known to increase fusel alcohol production, but to lower ester levels [51]–[53]. In our study, *S. cerevisiae* presented a down-regulation of acetyltransferase gene *AYT1* at 28°C, but high levels of higher alcohols and acetate esters suggest that acetate ester synthesis might be more dependent on substrate availability than on the expression of acetyltransferase genes. Nevertheless, this was not the case of *S. bayanus var. uvarum* at 28°C because low levels of acetate esters were found despite the high production for higher alcohols found.

*S. cerevisiae* presented the highest production of higher alcohols and up-regulations in most genes relating to higher alcohol production.The highest acetate ester producer at 12°C was *S. bayanus var. uvarum*, which showed an up-regulation in acetyltransferase gene *ATF2*, and despite presenting an up-regulation of the esterase *TIP1* gene, this esterase has been related only to ethyl esters. At 28°C, the main acetate ester producers were *S. kudriavzevii* and *S. cerevisiae* [26]. *S. kudriavzevii* presented an up-regulation in the *ATF2* gene, whereas *S. cerevisiae* showed a down-regulation of one acetyltransferase gene, which has not been related to acetate ester synthesis.

In conclusion, the transcriptome analysis of the genes related to aroma production can provide us with an idea of the compounds that will be synthesized during the fermentation process, as previously stated by other authors for *S. cerevisiae* [34]. Remarkable differences in the gene expression level were observed when comparing the three species, *S. cerevisiae*, *S. bayanus var. uvarum* and S. *kudriavzevii*, which resulted in different aroma profiles. Knowledge of these differences in the transcriptome can be a tool to help modulate aroma in order to create wines that offer the desired aromatic traits.
